# Validation of a Stability-Indicating RP-HPLC Method for the Simultaneous Determination of Trimethoprim and Sulfadimethoxine Sodium in Oral Liquid Dosage Form

**DOI:** 10.3797/scipharm.1212-30

**Published:** 2013-02-18

**Authors:** Mashhour M. Ghanem, Saleh A. Abu-Lafi

**Affiliations:** 1 Pharmacare Pharmaceutical Company, P.O. Box 677, Ramallah, Palestine.; 2 Faculty of Pharmacy, Al-Quds University, P.O. Box 20002, Abu-Dies, Palestine.

**Keywords:** Trimethoprim, Sulfadimethoxine sodium, Vetricine^®^ oral solution, Validation, Stability indicating method

## Abstract

A simple, specific, accurate, and stability-indicating RP-HPLC method was developed and validated for the simultaneous determination of Trimethoprim (TMP) and Sulfadimethoxine sodium (SDMS) in Vetricine^®^ oral solution product. The desired separation was achieved on an ODS column (250×4.6 mm i.d., 5 μm) at room temperature. The optimized mobile phase consisted of an isocratic solvent mixture of water:acetonitrile:triethylamine (700:299:1, v/v/v), adjusted to a pH of 5.7 ± 0.05 with 0.2N acetic acid. The mobile phase was fixed at 0.8 ml/min and the analytes were monitored at 254 nm using a photodiode array detector. The effects of the chromatographic conditions on the peaks USP tailing factor, column efficiency, and resolution were systematically optimized. Forced degradation experiments were carried out by exposing TMP, SDMS standards, and the oral solution formulation to thermal, photolytic, oxidative, and acid-base hydrolytic stress conditions. The degradation products were well-resolved from the main peaks and the excipients, thus proving the reliable stability-indicating method. The method was validated as per ICH and USP guidelines (USP34/NF29) and found to be adequate for the routine quantitative estimation of TMP and SDMS in commercially available Vetricine® oral liquid dosage form.

## Introduction

**Fig. 1:**
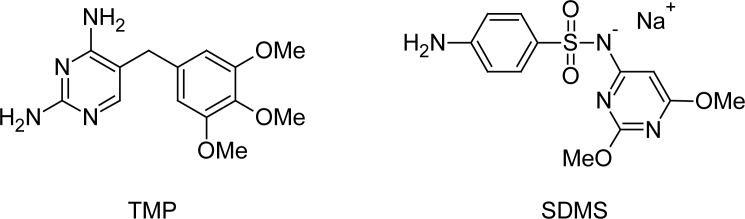
Chemical structures of TMP and SDMS

Vetricine^®^ oral solution is a veterinary drug that combines two antibacterial substances, trimethoprim (TMP) and sulfadimethoxine sodium (SDMS). It is a synthetic antimicrobial drug with broad spectrum bactericidal action. It is used for the treatment of infections caused by: Escherichia coli, Salmonella spp., Enterobacter spp., Klebsiella spp., Staphylococcus spp., Vibrio cholerae, Haemophilus influenzae; and other bacteria sensitive to TMP and SDMS especially in poultry and rabbits [1]. Figure 1 shows the chemical structure of the two active ingredients present in the Vetricine^®^ oral solution. TMP is official in BP and USP [2, 3], whereas SDMS is only official in the USP [4]. The combined simultaneous analysis of both drugs has not been adopted in any official pharmacopoeia.

Several HPLC procedures [5–9] and LC-MSMS methods [10–13] have been reported for the quantitative determination of TMP and SDMS, either alone or in combination. An extensive review of the literature revealed only one recent method that describes the simultaneous estimation of both drugs in pharmaceutical preparation [14]. This method was validated only for linearity, precision, accuracy, and specificity. Other parameters such as system suitability, robustness, sensitivity as manifested by limits of detection and limits of quantitation were not investigated. The name of the pharmaceutical preparation used and the placebo were never mentioned. Liquid preparations for the oral multidose solution usually contain excipients and suitable preservatives to minimize the risk of microbial contamination growth. Therefore, this method is not suitable for the routine quantification of TMP and SDMS in Vetricine^®^ oral solution. In the same paper [14], the extreme buffer of pH 2.0 was used which may shorten the lifetime of the column upon running extensive routine analysis. Moreover, the calculated capacity factor (k’) of TMP was about 0.5, while in practice, the k’ value should always be greater than one to prevent the overlap of TMP with any solvent system peaks. These shortcomings indicate that the published method is not satisfactory to carry out the routine analysis of Vetricine^®^ oral solution.

Therefore, there is a need to develop another new validated stability-indicating quality control method to allow the simultaneous determination of TMP and SDMS in the Vetricine^®^ oral solution. The proposed method is able to separate both drugs from one another, from the six unknown degradation products, and from the benzyl alcohol preservative. Subsequently, this method was validated as per ICH/USP guideline validation norms [15, 16].

## Results and Discussion

### Method development and Optimization

The main principle prior to the development of a proper RP-HPLC method was to be able to separate TMP and SDMS from the placebo, which includes a benzyl alcohol preservative, as well as to separate them from all degradation products. Moreover, the designed method should be simple enough to use for a routine quality control laboratory. Therefore, various mobile phases have been examined to achieve this specific target. Variables such as acetonitrile (ACN) strength, triethylamine (TEA) additive concentration, mobile phase pH, and temperature were investigated. The overlaid ultraviolet absorption spectra of the two active ingredients TMP and SDMS (0.05 mg/ml each) demonstrated that they shared a wavelength near 254 nm, which therefore was chosen for the entire study.

The method development process was initiated with a combination of water: acetonitrile (65:35; v/v) adjusted to a pH of 6.0 using 0.1N acetic acid. Both drugs peaks were very broad, particularly SDMS which had a tailing factor of more than 2.8. The presence of residual silanols on any type of silica-based stationary phase is known to pose tailing particularly for amines. Subsequently, different concentrations of 0.05%, 0.1%, and 0.15% TEA additive were tested to reduce band broadening of the drugs. It turns out that using 0.1% and 0.15% TEA made the peaks sharper, so the tailing factor value of SDMS decreased to 1.4. Therefore, 0.1% TEA additive concentration was chosen for the entire study. The effect of ACN strength at a fixed TEA concentration and pH was also explored. Different percentages of 20%, 30%, 40%, and 50% ACN were tried. At 20% ACN level, the retention time of SDMS was about 33 minutes, while at 40% ACN, TMP was eluted near the void peak. Therefore, 30% of ACN was selected as an optimal value.

**Fig. 2:**
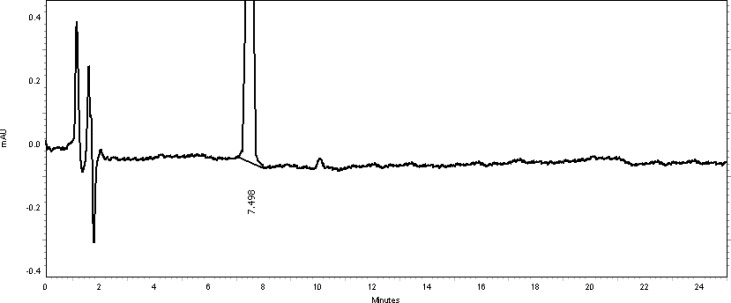
Zoomed-in view of a typical placebo chromatogram (water, polyethylene glycol 400, and benzyl alcohol). The peak at 7.498 minutes is due to benzyl alcohol preservative

**Fig. 3:**
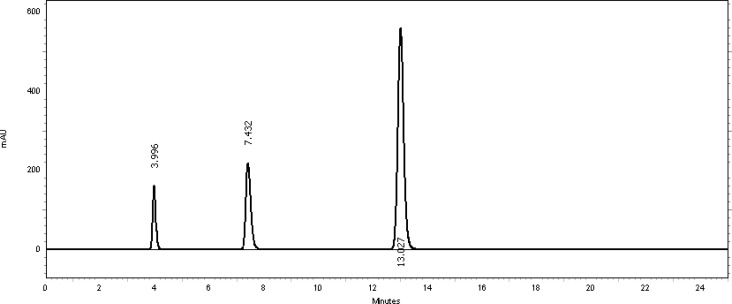
Typical chromatogram of a standard mixture of 40 μg/ml TMP (3.996 minutes), 213.6 μg/ml SDMS (13.027 minutes) and benzyl alcohol preservative (7.432 minutes).

The study of pH’s effect on resolution was deemed necessary to further optimize the separation conditions. The tested mobile phase pH’s were from 3.7 up to 7.7 at increments of 0.5. The best resolution and tailing factor with reasonable analysis time for both ingredients was accomplished at pH 5.7. Different temperatures of 15°C, 20°C, 25°C, 30°C, and 35°C were also evaluated. It was found that varying temperatures between 15°C and 35°C had no considerable influence on resolution or on the tailing factor values, and therefore room temperature was selected. Figure 2 shows a typical chromatogram of the placebo used which contains purified water, polyethylene glycol 400, and benzyl alcohol preservative at the optimized conditions. Figure 3 also shows a typical HPLC chromatogram of the freshly prepared mixture of TMP and SDMS and benzyl alcohol preservative using the optimized conditions.

### Method Validation

After the successful optimization of the RP-HPLC method, it was validated in accordance to the ICH/USP guidelines [15, 16]. Parameters such as system suitability, specificity (placebo and forced degradation interferences), sensitivity (LOD and LOQ), linearity, range, accuracy (recovery), precision (repeatability and intermediate precision), robustness, and stability-indicating capability were all validated.

### System suitability

The system suitability was determined by injecting six replicates of the standard solutions and analyzing each active ingredient for its peak area, peak USP tailing factor, resolution, number of theoretical plates, and capacity factor. The system suitability results for a combined solution of 40 μg/ml TMP and 213.6 μg/ml SDMS revealed %RSD of less than 1.0% for both peak areas. This method meets the accepted requirements as shown in Table 1.

### Specificity (placebo and forced degradation interference)

Generally, the specificity of a method is its suitability for the analysis of a compound in the presence of potential impurities. Placebo, standards, and sample test solutions were all injected at the same wavelength of 254 nm to assure the specificity of the optimized method. A comparison of the retention times of TMP and SDMS in sample solutions and in the standard solutions were exactly the same. Figures 2 and 3 showed that there were no interferences at the retention times for TMP and SDMS due to the placebo. Therefore, the proposed method is suitable for the quantification of the active ingredients in Vetricine^®^ oral solution.

The specificity of the method for TMP and SDMS has been determined in the presence of six stress impurities. It was assessed by performing forced degradation studies on pure standards of the active ingredients separately to indicate the initial results, and also on samples of Vetricine^®^ oral solution in the presence of their potential degradants. The stress conditions studied were UV light (254 nm), heat (70°C), acid hydrolysis (1.0 N HCl), base hydrolysis (1.0 N NaOH), and oxidation (10% H_2_O_2_). The sample stress solutions were analyzed against freshly prepared standards and samples. The assay and purity check (at 10% height) for the stressed standards and sample solutions were calculated as summarized in Table 2.

**Fig. 4:**
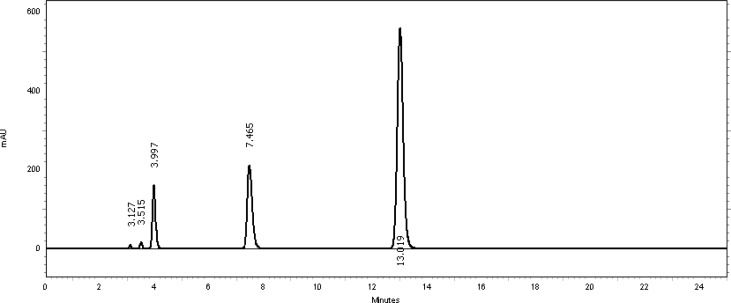
HPLC chromatogram of the Vetricine^®^ oral solution upon exposure to UV light for 48 hours, TMP (3.997 minutes), SDMS (13.019 minutes), benzyl alcohol (7.465 minutes). The two unknown degraded impurities appeared at 3.127 and 3.515 minutes.

**Fig. 5:**
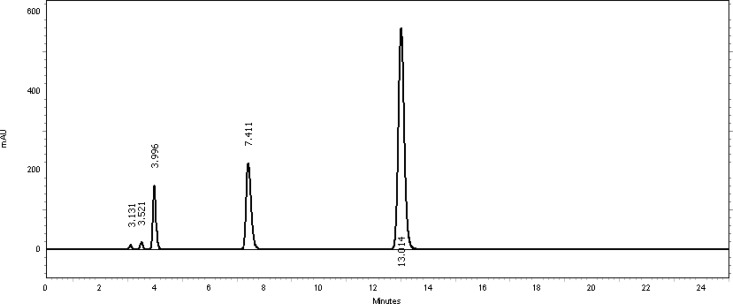
HPLC chromatogram of thermal degradation of the Vetricine^®^ oral solution upon exposure to heat for 72 hours, TMP (3.996 minutes), SDMS (13.014 minutes), benzyl alcohol (7.411 minutes). The two unknown degraded impurities appeared at 3.131 and 3.521 minutes.

**Fig. 6:**
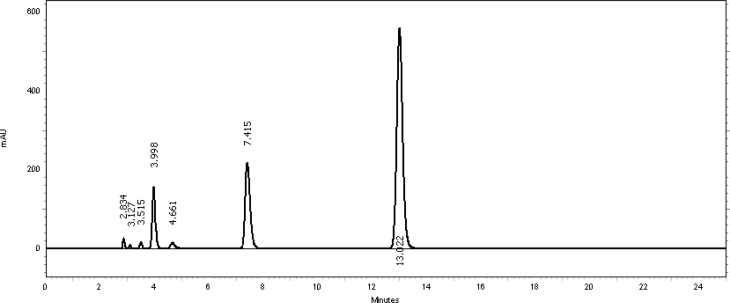
HPLC chromatogram of acidic degradation of the Vetricine^®^ oral solution after 60 minutes, TMP (3.998 minutes), SDMS (13.022 minutes), benzyl alcohol (7.415 minutes). The four unknown degraded impurities appeared at 2.834, 3.127, 3.515, and 4.661 minutes.

**Fig. 7:**
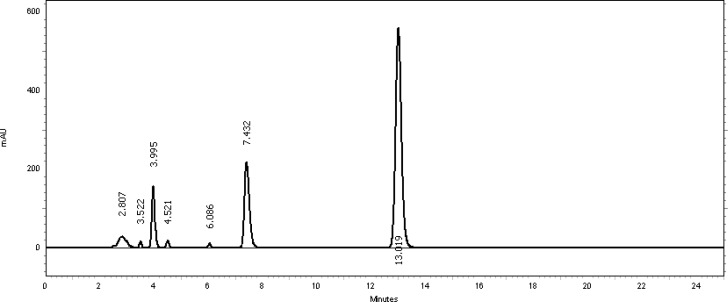
HPLC chromatogram of basic degradation of the Vetricine^®^ oral solution after 60 minutes, TMP (3.995 minutes), SDMS (13.019 minutes), benzyl alcohol (7.432 minutes). The four unknown degraded impurities appeared at 2.807, 3.522, 4.521, and 6.086 minutes.

**Fig. 8:**
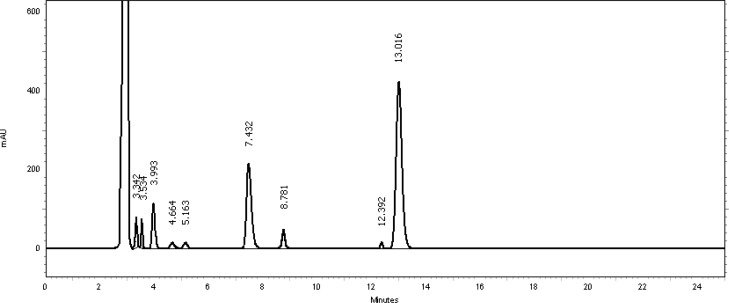
HPLC chromatogram of oxidative degradation of the Vetricine^®^ oral solution after 24 hours, TMP (3.993 minutes), SDMS (13.016 minutes), benzyl alcohol (7.432 minutes). The six unknown degraded impurities appeared at 3.342, 3.534, 4.664, 5.163, 8.781, and 12.392 minutes. The first eluted nonintegrated peak is due to H_2_O_2_.

Table 2 revealed that the oxidative stress results showed extensive degradation in comparison to other stress conditions. Peak purity index for both active ingredients was found to be no less than 0.9998, a higher value than the accepted limit (0.990). Therefore, there was no interference between the main active ingredients and any other stress impurity peaks in the chromatogram. Almost the same pattern of degradation was obtained for both TMP and SDMS in their Vetricine^®^ oral solution samples. Figures (4–8) show the chromatographic profiles of the active ingredients and the degradation products after exposing the Vetricine^®^ oral solution to different stress conditions as in Table 2.

### Sensitivity

The sensitivity of the method was explored via measurement of the limit of detection (LOD) and limit of quantitation (LOQ) for TMP and SDMS at a signal-to-noise ratio of 3 and 10, respectively. It was achieved by injecting a series of diluted solutions with known concentrations. The LOD was found to be 0.8 and 1.0 μg/ml for TMP and SDMS, respectively. The LOQ was found to be 2.7 and 3.3 μg/ml for TMP and SDMS, respectively, with RSD of 3.95% and 4.36% for TMP and SDMS, respectively (accepted value is less than 10%).

### Linearity and range

Different amounts of TMP and SDMS in the range of 60% to 140% of the labeled amount (five concentration levels and three replicates each) were spiked with the Vetricine^®^ matrix (water, polyethylene glycol 400, and benzyl alcohol preservative).

**Fig. 9:**
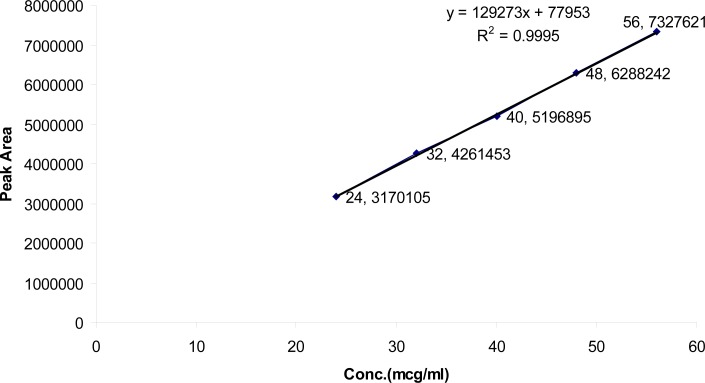
Linearity and range for TMP

**Fig. 10:**
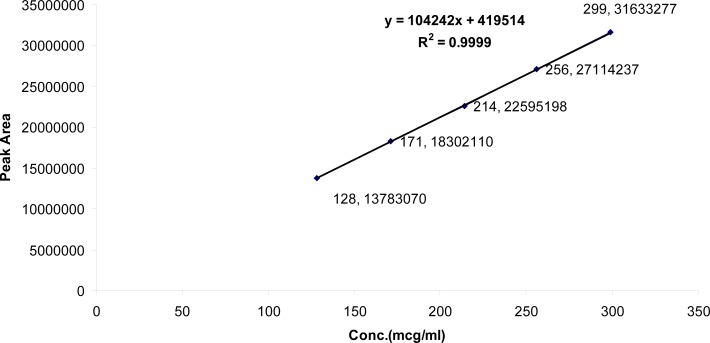
Linearity and range for SDMS

The linearity in the range of 24–56 μg/ml and 128–299 μg/ml for TMP and SDMS, respectively, was investigated. The regression lines demonstrated linearity in the tested range. The regression analysis confirmed that the deviation of the y-intercept from zero is not significant (less than 2%) in compliance with ICH and USP recommendations [15, 16]. The regression lines were linear with *R^2^* of 0.9995 and 0.9999 for TMP and SDMS, respectively (Figures 9 and 10).

### Accuracy (recovery)

Accuracy was determined by the recovery study of known amounts of TMP and SDMS standards added to a placebo matrix for oral dosage form. Different concentrations of the two active ingredients were added to the placebo matrix and the recovery was measured. The data obtained for the evaluation of linearity were used. The accuracy as reflected from recovery data and statistical evaluation of the assay for the two active ingredients is listed in Table 4. The average recovery data of TMP and SDMS showed results between 98.8% and 101.7% with % RSD of less than 1.1%, which are within acceptable limits (98.0 to 102.0% recovery and %RSD of not more than 2.0%).

### Precision

#### Repeatability

One laboratory analyst carried out the assay of TMP and SDMS on six determinations of a homogeneous sample of Vetricine^®^ oral solution at 100% level of the test concentration with the same analytical equipment on the same day. The assay results and statistical evaluation for the assay of the two active ingredients showed %RSD values of 0.94% and 0.86% for TMP and SDMS, respectively, which are within the acceptable limit of 2.0%.

#### Intermediate Precision (ruggedness)

Two laboratory analysts carried out the assay of TMP and SDMS on 12 homogeneous samples of Vetricine^®^ oral solution at 100% level of the final test concentration with two different sets of analytical equipment on two different days. The assay results and statistical evaluation for the assay of the two active ingredients revealed % RSD values of 1.36% and 1.18% for TMP and SDMS, respectively, which are within the acceptable limit of 2.0%. The results of the assay of the two ingredients proved that the method is repeatable and rugged enough for day-to-day use.

#### Robustness

Predetermined variations were performed under the experimental conditions of the RP-HPLC method to assess its robustness. The six variations imposed on the chromate-graphic method are summarized in Table 5. The modifications include different mobile phase flow rates of 0.7, 0.8, and 0.9 ml/min and three different column temperatures in the range 15–35°C. Different TEA percentages in the mobile phase (in the range of ± 5 of the nominal value and the normal % TEA) and different ACN percentages in the mobile phase (in the range of ± 5 of the nominal value and the normal % ACN) were also investigated. Three column batches filled with the same prescribed stationary phases were studied. Finally, three different pH values of the mobile phase at 5.5, 5.7, and 5.9 were tested. The % RSD values showed no significant change in the final assay results of each of the above two ingredients using the six variations (Table 5).

### Applicability of the method to marketed products:

It is evident from the results obtained that the validated method gave satisfactory results with respect to the analysis of both drugs. The validated method is applied to a commercially available package (Vetricine^®^ oral solution) as shown in Table 6.

This acceptable value indicated the applicability of the proposed method for the routine quality control of Vetricine^®^ oral solution without interference from the excipients or the preservatives. This was evidenced by the good labeled claim percentages as well as the absence of any peaks in the chromatogram of the oral solution.

## Experimental

### Materials

Reference standards of trimethoprim (TMP) and sulfadimethoxine sodium (SDMS) were purchased from Sigma-Aldrich (Germany). Glacial acetic acid, triethylamine (TEA), HPLC grade acetonitrile (ACN), and methanol (MeOH) solvents, hydrochloric acid fuming (37%), sodium hydroxide pellets, and hydrogen peroxide (30%), were purchased from Merck (Germany). Highly purified water was prepared by using a Millipore Milli-Q Plus water purification system. Vetricine^®^ oral solution (labeled claim: each one ml contains 25 mg TMP and 133.5 mg SDMS) samples, and all of the active ingredients and excipients usually used in manufacturing the pharmaceutical combination, were kindly supplied by Pharmacare pharmaceutical company, Palestine.

### HPLC system

The HPLC system consisted of LaChrom (Merck-Hitachi) equipped with a model L-7100 pump, L-7200 autosampler, L-7300 column oven, DAD L-7450 photodiode array (PDA) detector, and D-7000 software HSM version 3.1 (Merck Hitachi, England). A double beam ultraviolet-visible spectrometer (PG Instruments, United Kingdom) was used.

### Chromatographic conditions

The HPLC experimental conditions were optimized on the Octadecyl Silane C18 chemically bonded column (250 mm × 4.6 mm i.d., 5 μm particles) that was purchased from ACE, United Kingdom. The optimum mobile phase was prepared by mixing water with ACN and TEA (700:299:1; v/v/v), and then adjusted to a pH of 5.7 ± 0.05 with 0.2 N glacial acetic acid. The mobile phase was filtered by using a 0.45 μm microporous filter and was degassed by sonication prior to use. A wavelength of 254 nm was chosen since it was found to be the most appropriate for the determination of the two active ingredients. The flow rate used was 0.8 ml/minute. The injection volume was 20 μl and the temperature of the column was room temperature. The total run time of the last eluted SDMS was about 14 minutes.

### Preparation of standard solutions

The standard solution for both drugs was prepared by dissolving 25 mg TMP reference standard and 133.5 mg SDMS reference standard in 35 ml of MeOH, shaken by mechanical means for five minutes, sonicated for two minutes, and then diluted up to 50 ml with the same solvent. Using a volumetric pipette, 2 ml of this solution was transferred to a 25 ml volumetric flask and completed to the volume using the mobile phase. This solution was filtered using a 0.45 μm membrane filter before analysis. The obtained final solution contained 40μg/ml TMP and 213.6μg/ml SDMS. This solution was directly protected from light.

### Preparation of sample solution

One ml of commercial Vetricine^®^ oral solution was transferred to a 50 ml volumetric flask containing 35 ml of MeOH, shaken by mechanical means for five minutes, sonicated for two minutes, and then diluted up to 50 ml with the same solvent. Using a volumetric pipette, 2 ml of this solution was transferred to a 25 ml volumetric flask and completed to the volume using the mobile phase. This solution was filtered using a 0.45 μm membrane filter before analysis. The obtained final solution contained 40μg/ml TMP and 213.6μg/ml SDMS. This solution was directly protected from light.

### Forced degradation study

ICH prescribed stress conditions such as acidic, basic, oxidative, thermal, and photolytic stresses, which were carried out.

### Standard drug stock solutions

The forced degradation study was conducted on solutions that were prepared by transferring 25 mg TMP reference standard into five different 50 ml volumetric flasks. Also, 133.5 mg SDMS reference standards were transferred separately into another five different 50ml volumetric flasks. Then 35 ml of MeOH was added to each flask and shaken by mechanical means for five minutes, and sonicated for two minutes until completely dissolved. These stock solutions were kept at room temperature protected from light and used for forced degradation studies.

### Acid hydrolysis

Five ml of 1.0 N HCl was added to one of the flasks containing the TMP stock solution and another 5 ml was added into one of the flasks containing the SDMS stock solution, kept at room temperature for 60 minutes in a dark place, and then diluted to 50 ml with MeOH. Two ml of this solution was transferred to a 25 ml volumetric flask, neutralized with 0.1 N NaOH, and completed to the volume using the mobile phase. This solution was filtered using a 0.45 μm membrane filter before analysis. The obtained final solution contained 40 μg/ml TMP and 213.6 μg/ml SDMS.

### Base hydrolysis

Five ml of 1.0 N NaOH was added to one of the flasks containing the TMP stock solution and another 5 ml was added to one of the flasks containing the SDMS stock solution, kept at room temperature for 60 minutes in a dark place, and then diluted to 50 ml with MeOH. Two ml of this solution was transferred to a 25 ml volumetric flask, neutralized with 0.1 N HCl, and completed to the volume using the mobile phase. This solution was filtered using 0.45 μm membrane filter before analysis. The obtained final solution contained 40 μg/ml TMP and 213.6 μg/ml SDMS.

### Oxidative hydrolysis

Ten ml of 10% H_2_O_2_ was added to one of the flasks containing the TMP stock solution and another 10 ml was added to one of the flasks containing the SDMS stock solution, kept at room temperature for 24 hours in a dark place, and then diluted to 50 ml with MeOH. Two ml of this solution was transferred to a 25 ml volumetric flask and completed to the volume using the mobile phase. This solution was filtered using a 0.45 μm membrane filter before analysis. The obtained final solution contained 40μg/ml TMP and 213.6μg/ml SDMS.

### Thermal degradation

One of the flasks containing the TMP stock solution and another one containing the SDMS stock solution were studied separately for their thermal degradation by keeping them at 70°C in a water bath, protected from light for 72 hours, and then diluted to 50 ml with MeOH. Two ml of this solution was transferred into a 25 ml volumetric flask and completed to the volume using the mobile phase. This solution was filtered using a 0.45 μm membrane filter before analysis. The obtained final solution contained 40μg/ml TMP and 213.6μg/ml SDMS.

### Photo degradation

One of the flasks containing the TMP stock solution and another one containing the SDMS stock solution were studied separately for their photodegradation by exposing them to UV light at 254 nm for 48 hours and then diluting them to 50 ml with MeOH. Two ml of this solution was transferred into a 25 ml volumetric flask and completed to the volume using the mobile phase. This solution was filtered using a 0.45 μm membrane filter before analysis. The obtained final solution contained 40μg/ml TMP and 213.6μg/ml SDMS.

### Forced degradation study on Vetricine^®^ oral solution

The sample stock solutions were prepared by separately transferring 1 ml of the Vetricine^®^ oral solution (containing 25 mg TMP and 133.5 mg SDMS) into a series of five different 50 ml volumetric flasks. The very same procedure adopted for the standard solutions was used in the Vetricine^®^ oral solution. The obtained final solution contained 40μg/ml TMP and 213.6μg/ml SDMS.

## Conclusion

The validated HPLC method developed for the quantitative quality control determination of TMP and SDMS in Vetricine^®^ oral solution was evaluated for system suitability, specificity, sensitivity, linearity, range, accuracy (recovery), precision (repeatability and intermediate precision), and robustness. All the validation results were within the allowed specifications of ICH/USP guidelines. The developed method has proven to be rapid, accurate, and stability-indicating for the simultaneous determination of the combined TMP and SDMS in Vetricine^®^ oral solution in the presence of excipients and the degradation products. There was always a complete separation of both ingredients from their degradation products and from the placebo. As a result, the proposed HPLC method could be adopted for the quantitative quality control and routine analysis of Vetricine^®^ oral solution or any other formulation.

## Figures and Tables

**Tab. 1.** Summary of the accepted system suitability requirements

**Parameter**	**TMP**	**SDMS**	Accepted limit*
% RSD	0.87	0.64	≤ 2.0%
Tailing factor (T_f_)	1.18	1.29	≤ 2.0
Resolution (R_s_)	–	13.8	≥2.0
Number of theoretical plates (N)	8342	12034	≥3000
Capacity factor (k’)	1.3	6.4	≥1.0

* Set according to Palestinian Ministry of Health Registration Department criteria.

**Tab. 2.** Summary of the forced degradation of TMP and SDMS standards and Vetricine^®^ oral solution

**Name**	**Stress condition**	**Degradation%**	Purity index*
**TMP standard**	Acidic/1.0 N HCl / 60 min at RT	7.21	1.0000
	Alkaline/1.0 N NaOH / 60min at RT	6.06	0.9999
	Oxidative/10 % H_2_O_2_ /24 hours at RT	23.27	1.0000
	Thermal/70 °C/72 hours	4.13	1.0000
	Light/ UV-254nm /48 hours	3.22	1.0000

**TMP sample**	Acidic/1.0 N HCl / 60 min at RT	7.08	1.0000
	Alkaline/1.0 N NaOH / 60min at RT	5.98	0.9999
	Oxidative/10 % H_2_O_2_ /24 hours at RT	23.54	0.9998
	Thermal/70 °C/72 hours	4.07	1.0000
	Light/ UV-254nm /48 hours	3.13	1.0000

**SDMS standard**	Acidic/1.0 N HCl / 60 min at RT	5.73	1.0000
	Alkaline/1.0 N NaOH / 60min at RT	5.13	1.0000
	Oxidative/10 % H_2_O_2_ /24 hours at RT	21.98	1.0000
	Thermal/70 °C/72 hours	3.54	1.0000
	Light/ UV-254nm /48 hours	3.02	1.0000

**SDMS sample**	Acidic/1.0 N HCl / 60 min at RT	5.64	1.0000
	Alkaline/1.0 N NaOH / 60min at RT	5.13	1.0000
	Oxidative/10 % H_2_O_2_ /24 hours at RT	21.86	1.0000
	Thermal/70 °C/72hours	3.41	1.0000
	Light/ UV-254nm /48 hours	3.04	1.0000

* The accepted criteria is > 0.990.

**Tab. 3.** Regression statistics

**Active ingredient**	**Linearity range (μg/ml)**	**(R^2^)**	Linearity equation*	**Y-intercept (%) %**
TMP	24–56	0.9995	Y = 129273X + 77953	1.499%
SDMS	128–299	0.9999	Y = 104242X + 419514	1.857%

* Y is the dependent variable and X is the independent variable.

**Tab. 4.** Average recoveries, % RSD values at five concentration levels of spiking of TMP and SDMS

**Active ingredient**	**Amount added (level %)**	**Average recovery (%) ± S.D *(n=3)***	**RSD (%) *(n=3)***
**TMP**	24 μg /ml (60%)	100.9 ± 0.64	0.63
	32 μg /ml (80%)	101.7 ± 0. 82	0.81
	40 μg /ml (100%)	99.2 ± 0.77	0.78
	48 μg /ml (120%)	100.0 ± 1.05	1.05
	56 μg /ml (140%)	99.9 ± 0.93	0.93

**SDMS**	128 μg/ml (60%)	100.5 ± 0.96	0.96
	171 μg/ml (80%)	100.1 ± 0.91	0.91
	214 μg/ml (100%)	98.9 ± 0.86	0.87
	256 μg/ml (120%)	98.8 ± 1.07	1.08
	299 μg/ml (140%)	98.9 ± 0.77	0.78

**Tab. 5.** Robustness testing of the two active ingredients of TMP and SDMS

**Active ingredient**	**Parameter**	**Average assay% ± S.D *(n=3)***	**Average RT(min) ±S.D. *(n=3)***
**TMP**	0.7ml/min flow	98.5 ± 0.56	4.491 ± 0.016
	0.8ml/min flow	98.6 ± 0.58	3.997 ± 0.014
	0.9ml/min flow	99.7 ± 1.39	3.498 ± 0.011
	28.4% ACN	101.6 ± 0.43	4.194 ± 0.018
	29.9% ACN	101.5 ± 0.47	4.009 ± 0.016
	31.4% ACN	100.4 ±1.42	3.837 ± 0.014
	Temperature (°C)	99.7 ± 0.87	4.007 ± 0.054
	% TEA buffer	99.3 ± 1.22	4.003 ± 0.058
	Column batches	100.3 ±1.03	3.998 ± 0.031
	Mobile phase pH	99.6 ± 0.84	3.995 ± 0.061

**SDMS**	0.7ml/min flow	100.3 ± 0.91	14.793 ± 0.048
	0.8ml/min flow	101.1 ± 0.95	13.029 ± 0.041
	0.9ml/min flow	100.9 ± 0.42	11.574 ± 0.038
	28.4% ACN	101.5 ± 0.30	14.107 ± 0.046
	29.9% ACN	100.2 ± 0.53	13.018 ± 0.039
	31.4% ACN	99.4 ± 0.44	12.016 ± 0.035
	Temperature (°C)	99.7 ± 1.16	13.029± 0.193
	% TEA buffer	98.9 ±1.31	13.031± 0.124
	Column batches	101.4 ± 0.97	13.042± 0.075
	Mobile phase pH	99.3 ±1.27	13.022± 0.134

**Tab. 6.** Result of market product (Vetricine^®^ oral solution)

**Product Name**	**Labeled claim (mg/ml)**	**TMP (mg/ml)**	**SDMS (mg/ml)**
Vetricine® oral solution	TMP(25), SDMS(133.5)	25.4	135.2
